# Association Between Changes in Serum and Skeletal Muscle Metabolomics Profile With Maximum Power Output Gains in Response to Different Aerobic Training Programs: The Times Study

**DOI:** 10.3389/fphys.2021.756618

**Published:** 2021-10-20

**Authors:** Alex Castro, Renata G. Duft, Silas Gabriel de Oliveira-Nunes, André L. L. de Andrade, Claudia R. Cavaglieri, Mara Patricia Traina Chacon-Mikahil

**Affiliations:** ^1^Laboratory of Exercise Physiology, School of Physical Education, University of Campinas (UNICAMP), São Paulo, Brazil; ^2^Nuclear Magnetic Resonance Laboratory, Department of Chemistry, Federal University of São Carlos (UFSCar), São Paulo, Brazil; ^3^School of Medical Sciences, University of Campinas (UNICAMP), São Paulo, Brazil

**Keywords:** trainability, cardiorespiratory fitness, metabolomics, metabolites, responsiveness

## Abstract

**Purpose:** High heterogeneity of the response of cardiorespiratory fitness (CRF) to standardized exercise doses has been reported in different training programs, but the associated mechanisms are not widely known. This study investigated whether changes in the metabolic profile and pathways in blood serum and the skeletal muscle are associated with the inter-individual variability of CRF responses to 8-wk of continuous endurance training (ET) or high-intensity interval training (HIIT).

**Methods:** Eighty men, young and sedentary, were randomized into three groups, of which 70 completed 8 wk of intervention (> 90% of sessions): ET, HIIT, or control. Blood and vastus lateralis muscle tissue samples, as well as the measurement of CRF [maximal power output (MPO)] were obtained before and after the intervention. Blood serum and skeletal muscle samples were analyzed by 600 MHz ^1^H-NMR spectroscopy (metabolomics). Associations between the pretraining to post-training changes in the metabolic profile and MPO gains were explored via three analytical approaches: (1) correlation between pretraining to post-training changes in metabolites' concentration levels and MPO gains; (2) significant differences between low and high MPO responders; and (3) metabolite contribution to significantly altered pathways related to MPO gains. After, metabolites within these three levels of evidence were analyzed by multiple stepwise linear regression. The significance level was set at 1%.

**Results:** The metabolomics profile panel yielded 43 serum and 70 muscle metabolites. From the metabolites within the three levels of evidence (15 serum and 4 muscle metabolites for ET; 5 serum and 1 muscle metabolites for HIIT), the variance in MPO gains was explained: 77.4% by the intervention effects, 6.9, 2.3, 3.2, and 2.2% by changes in skeletal muscle pyruvate and valine, serum glutamine and creatine phosphate, respectively, in ET; and 80.9% by the intervention effects; 7.2, 2.2, and 1.2% by changes in skeletal muscle glycolate, serum creatine and creatine phosphate, respectively, in HIIT. The most changed and impacted pathways by these metabolites were: arginine and proline metabolism, glycine, serine and threonine metabolism, and glyoxylate and dicarboxylate metabolism for both ET and HIIT programs; and additional alanine, aspartate and glutamate metabolism, arginine biosynthesis, glycolysis/gluconeogenesis, and pyruvate metabolism for ET.

**Conclusion:** These results suggest that regulating the metabolism of amino acids and carbohydrates may be a potential mechanism for understanding the inter-individual variability of CRF in responses to ET and HIIT programs.

## Introduction

Physical inactivity and low levels of cardiorespiratory fitness (CRF) are currently considered a threat to public health. As a consequence, international agencies recommend that adults accumulate ≥150 min·wk^−1^ of moderate-intensity cardiorespiratory exercise training, ≥75 min·wk^−1^ of vigorous-intensity cardiorespiratory exercise training, or a combination of moderate- and vigorous-intensity exercise to achieve a total energy expenditure of ≥500–1000 MET·wk^−1^, in an attempt to minimize the occurrence of non-communicable diseases (Garber et al., 2011; Riebe et al., 2018). However, several previous investigations demonstrate a wide variability of individual responses to standardized doses of exercise. There is a substantial number of individuals who do not show clinically important increases in CRF after completing traditional continuous endurance training (ET) or alternative high-intensity interval training (HIIT) programs conducted under current recommendations for physical activity practices (Bouchard and Rankinen, 2001; Ross et al., 2015a; Williams et al., 2019, 2021; Bonafiglia et al., 2021).

In the past decade, with the technological advance of the comprehensive methods that make up the omic sciences (Tanaka et al., 2016; Kelly et al., 2020) new opportunities to investigate the integrative mechanisms of the variability of individual CRF responses to exercise have emerged (Sanford et al., 2020). So far, there are few genomic (Bouchard et al., 2011; Sarzynski et al., 2017; Williams et al., 2021), proteomic (Robbins et al., 2021), transcriptomic (Timmons et al., 2010; Keller et al., 2011; Dias et al., 2015) and metabolomic (Lewis et al., 2010; Huffman et al., 2014; Castro A., et al., 2019) studies involving the use of omic methods to study the variability of responses to exercise.

Particularly, metabolomics is a powerful metabolic phenotyping technology that allows to identify and quantify metabolites that reflect the biochemical activity underlying different physiological conditions and complex phenotypes (Rinschen et al., 2019; Wishart, 2019a), such as CRF (Castro A., et al., 2019; Castro et al., 2021). In this sense, nuclear magnetic resonance (NMR) spectroscopy is known for its reproducibility, nondestructive nature and minimal sample preparation (Wishart, 2019b), being one of the most widely employed metabolomics platforms for detecting and quantifying metabolites and their metabolic pathways related to exercise, physical activity, and health (Duft et al., 2017b; Kelly et al., 2020). Based on a metabolomic approach, Huffman et al. identified, in different training programs, that the improvement in CRF is associated with changes in the concentrations of acetyl-heavy chains and intermediates of the citric acid cycle in the skeletal muscle, accompanied by changes in the expression of genes related to the pathways of production of these metabolites (Huffman et al., 2014). More recently, Castro et al. showed a panel of baseline serum and skeletal muscle metabolites associated with inter-individual response variability of CRF (TIMES Study), suggesting involvement of amino acid metabolism and translation processes in ET and HIIT programs, and carbohydrate metabolism in ET program (Castro A., et al., 2019).

Although the study of the variability of responses to training has received some attention in the last 4 decades (Bouchard et al., 1980, 2011; Lortie et al., 1984; Bouchard and Rankinen, 2001; Kohrt et al., 2004; Ross et al., 2019, 2015a; Castro A., et al., 2019; Williams et al., 2019; Bonafiglia et al., 2021; Meyler et al., 2021), as we know, the metabolic determinants of inter-individual variability of CRF in response to different aerobic training programs, especially under an integrated view of the adaptations in blood serum and skeletal muscle tissue, remain largely unknown. Understanding through which metabolic pathways individuals improve CRF in different aerobic training programs may be useful to guide new studies on the mechanisms related to the variability of CRF responses and pave the way for novel CRF-enhancing strategies in exercise training routine. Therefore, the aim of this study was to investigate whether changes in the metabolic profile and metabolic pathways of blood serum and skeletal muscle tissue are associated with the inter-individual variability of CRF responses to ET and HIIT programs.

## Methods

The sample, study design, and exercise training protocol of the TraInability and MEtabolomicS study (TIMES study) have been described in details previously (Castro A., et al., 2019).

### Participants

A total of eighty healthy and sedentary young Caucasian men, which seventy were defined as completers [age: 23.7 ± 3.0 yr; height: 1.74 ± 0.06 m; body mass: 75.2 ± 8.8; body fat: 20.0 ± 7.4%; body mass index (BMI): 24.8 ± 2.5 kg·m^−2^], from the TIMES study, were used for analysis (Castro A., et al., 2019). Briefly, participants were sedentary and did not engage in regular exercise defined as 30 min.wk^−1^ at an energy expenditure of 6 METS or more in the previous 4 months (Riebe et al., 2018; Castro A., et al., 2019; Castro et al., 2021). All participants were free from diabetes (fasting glucose > 7.0 mmol·L−1), hypertension (blood pressure > 140/90 mmHg), dyslipidemia (based on medication use), severe obesity (defined as body mass index > 33 kg·m^−2^), smoking, metabolic disorders, heart diseases, musculoskeletal problems interfering with exercise or significant chronic respiratory conditions (Castro A., et al., 2019).

Written informed consent was obtained from each participant. The study was approved by the University's Research Ethics Committee (Number: 2.717.688; CAAE: 52997216.8.0000.5404) and included in the Brazilian Clinical Trials Registry (ensaiosclinicos.gov.br; RBR-3rh38g).

### Study Design

Prior to the intervention, blood and vastus lateralis muscle tissue samples were obtained. These evaluations were preceded by 12 h of fasting following a standardized meal. After 72 h, the body composition was evaluated (full body plethysmography), followed by cardiorespiratory assessment and retest 48 h later (Skinner et al., 1999). Seventy-two h after the last pre-training evaluation, 80 participants were randomized into three groups, with a 3:3:1 allocation ratio, and of the total sample, 70 completed the eight wk of intervention (exercise adherence > 90%): ET (*n* = 30), HIIT (*n* = 30) and Control (CO, *n* = 10). This unequal randomization strategy was used to ensure adequate sample size for correlation analysis and subsequent analysis of high and low responders in the intervention groups. After the 4th wk of intervention, cardiorespiratory assessment was performed to adjust the training's intensity. In the end, after 48 h of the last training session, the assessments referred to in the pre-training moment were repeated (Castro A., et al., 2019).

### Standardization of Meals Prior to Data Collection

The night before the blood collections and muscle biopsies (12 h before), the participants consumed a standardized and balanced meal (60% carbohydrate, 25% lipid and 15% protein), with an energy value corresponding to 30% of the total individual energy expenditure estimated in order to avoid effects of dietary variations on the metabolic profile of the blood and muscle tissue samples (Peake et al., 2014; Shrestha et al., 2015; Castro et al., 2020).

### Blood and Muscle Tissue Samples

The venous blood and muscle tissue samples were collected between 7 am and 10 am. After the blood samples' collection, they were kept at rest in a serological tube for 30 min, and then centrifuged at 5000 rpm for 10 min. Afterwards, the serum aliquots were obtained and stored in a freezer at −80°C. Following the blood collections, tissue biopsies of the dominant lower limb's vastus lateralis muscle were performed according to the procedure described above (Shanely et al., 2014). Prior to the tissue's extraction, the area was shaved and cleaned with an antiseptic. A small area over the selected region was anesthetized with 2% xylocaine, injected subcutaneously. After anesthesia, a small incision (~5 mm) was made up to the muscle fascia using a surgical scalpel. The biopsy needle was then inserted into the muscle (~3 cm) to obtain the sample. After the tissues' removal, the incision was closed and covered with bandages. After extraction, all samples were cleaned (free of blood and excess connective tissue), aliquoted, immediately frozen in liquid nitrogen, and stored at −80°C for further analysis.

### Body Composition Assessment

The participants were instructed to drink only water and not to consume food or exercise 2 h prior to the assessment. For plethysmography measurements, the participants were asked to wear only trunks and a shower cap, without shoes and metallic accessories. Body mass and height were measured using a digital scale and stadiometer (BOD POD, Cosmed, Chicago, USA), respectively. Body density was then assessed using a full-body plethysmograph calibrated according to the manufacturer's recommendations (BOD POD^®^ Body Composition System; Life Measurement Instruments; Concord, CA) (McCrory et al., 1995). In all evaluations, the ambient temperature and the humidity conditions were maintained between 20–22°C and ~60%, respectively, without significant variations in atmospheric pressure. Based on these data, body density was converted to fat percentage using the Siri equation (Siri, 1993).

### Cardiorespiratory Assessment

Cardiorespiratory assessment was performed during an incremental test until exhaustion using a cycle ergometer with electromagnetic braking (Corival 400, Quinton® Instrument Co., Groningen, Holland). Heart rate (HR) was continuously monitored by a cardio-frequency monitor (S810, Polar, Keple, Finland). The subjective perception of effort was recorded at the final 15 s, using the 6–20 Borg scale (Borg and Linderho, 1967). Before and after each test, the cycle ergometer was calibrated according to the manufacturer's recommendations. After 5 min of rest on the ergometer cycle, the incremental test started, with a 3-min warmup at 50 W, followed by 25 W.min^−1^ increments (Buchfuhrer et al., 1983). The pedaling cadence was maintained between 70−80 rpm. The test was interrupted when the participant was unable to continue and/or did not maintain a minimum cadence of 70 rpm despite verbal encouragement (Thompson et al., 2013).

The resting HR values were estimated from the average of the values recorded during the 5-min rest (Swain and Leutholtz, 1997). The maximum HR (HR_MAX_) was defined as the mean value in the test's final 10 s. The reserve HR (HRR) was estimated by subtracting the values at rest from the respective maximum values achieved in the incremental test (Swain and Leutholtz, 1997; Lounana et al., 2007). MPO was estimated as W+[25·(*t*/60)], where W is the last load reached and *t* is the number of seconds in the test's final load (Kuipers et al., 1985). The highest MPO value recorded between tests was considered for the analyses and defined as the measure of CRF (Castro A., et al., 2019; Castro et al., 2021). The within-test coefficient of variations (CV) and intraclass correlation coefficient (ICC) were 2.8% and 0.98, respectively.

Additionally, an incremental validation step was conducted based on the HR_MAX_ achieved in relation to the HR_MAX_ expected, considering all the tests performed by the participants. As previously reported, the intra-participant standard deviation for HR_MAX_ derived from repeated measures is expected to be around 4 bpm (Skinner et al., 1999). Thus, the cutoff value corresponding to twice the intra-participant standard deviation, or 8 bpm, was used as a validation criterion. In the case of the pre-training tests, the highest MPO was retained as reference. In the post-training moment, for the tests to be validated and the participant considered for further analysis, the HR_MAX_ achieved could not have been 8 bpm higher than the HR_MAX_ obtained in the pre-training test. When this criterion was not met, the participant was excluded from the study (Castro A., et al., 2019).

### Training Protocol

Throughout the training program, the exercise intensity was individualized and customized for each participant based upon HRR. Both ET and HIIT programs were designed to obtain the same exercise volume in total and by session. A complete and detailed description of training volume balancing between groups can be found elsewhere (Castro A., et al., 2019).

The training program was carried out on an ergometer cycle (U1x, Matrix, Brazil), with 40 min per session, for eight wk, divided into Stage 1 (first four wk) and Stage 2 (last four wk). At the end of Stage 1, a cardiorespiratory assessment was performed to adjust the training intensity to be prescribed in Stage 2. For ET, the participants exercised for 40 min at 70% of HRR, three times a wk, in Stage 1; and for 40 min at 75% of HRR, four times a wk, in Stage 2. For HIIT, the participants exercised for 40 min, with 5 min at 50% of HRR, followed by five 4-min intervals at 90% of HRR (effort phase) interspersed with 3-min intervals at 50% of HRR (recovery phase), three times a week, in Stage 1; and 5 min at 60% of HRR, followed by five 4-min intervals at 90% of HRR interspersed with 3-min intervals at 60% of HRR, four times a week, in Stage 2. For the control, the participants were instructed not to perform physical exercises for eight wk. After four wk, the control participants were contacted again to remind them about the importance of remaining sedentary and to schedule the tests for the end of the eight-wk period.

All training sessions were supervised by an experienced professional to ensure that the target HR and pedaling cadence (70–80 rpm) were maintained. All training sessions were carried out in a reserved environment, with temperature controlled between 21–23°C.

### Preparation of Blood Samples for Metabolomics

Prior to the analysis of the blood samples, 3 kDa filters (Amicon Ultra) were washed with 500 μl of Milli-Q H_2_O, followed by centrifugation at 14,000 rpm and 4°C for 10 min. After the fifth wash, spin was performed (inversion of the filter and rotation at 8,000 rpm for 5 s) to eliminate any remnants of Milli-Q H_2_O. Subsequently, 500 μl of blood serum were added to the filter and centrifuged at 14,000 rpm and 4°C for 45 min. After this period, the filtered serum (250 μl) were diluted in an deuterium oxide solution (290 μl D_2_O, 99.9 %; Cambridge Isotope Laboratories Inc., Massachusetts, USA) containing a phosphate buffer (60 μl, Monobasic Sodium Phosphate, NaH_2_PO_4_ – H_2_O-137.99 g/mol; Dibasic Sodium Phosphate, Na_2_HPO_3_ – 141.96 g/mol; 0.1 M, pH 7.4), 0.5 TMSP-d4 (3-(trimethylsilyl)-2,2′,3,3′-tetradeuteropropionic acid from Sigma-Aldrich), and added to a 5 mm NMR tube (Wilmad Standard Series 5 mm, Sigma-Aldrich®) for immediate acquisition of the spectra on the spectrometer (Duft et al., 2017a; Castro A., et al., 2019).

### Preparation of Muscle Tissue Samples for Metabolomics

The muscle tissue samples were processed following the Le Belle protocol (Belle et al., 2002) adapted by Castro et al. (Castro A., et al., 2019). Firstly, the samples (~40 mg) were weighed and added to a cold methanol/chloroform solution (2:1 v/v, total of 2.5 ml), after which they were homogenized on ice (3 × 30-s repetitions, interspersed with 10-s pauses) and sonicated for 3 min, with 10-s pauses between minutes. Subsequently, a cold chloroform/Milli-Q water solution (1:1 v/v, total of 2.5 ml) was added to the samples, which were then briefly stirred (to form an emulsion) and centrifuged at 4°C for 30 min (2000 *g*). The top phase of the mixture (methanol, water and polar metabolites) was collected and completely dried in a vacuum concentrator (miVac Duo Concentrator, Genevac, UK). The remaining solid phase was rehydrated in 0.6 ml deuterium oxide containing phosphate buffer (0.1 M, 7.4 pH) and 0.5 mM of TMSP-d4. Finally, the samples were added to a 5 mm NMR tube (Wilmad Standard Series 5 mm, Sigma-Aldrich®) for immediate acquisition of the spectra on the spectrometer.

### Acquisition of Spectra and Quantification of Metabolites

To obtain and quantify the metabolites via metabolomics, the spectra were acquired from the serum and skeletal muscle tissue samples at the National Biosciences Laboratory (LNbio – http://lnbio.cnpem.br/) using the VnmrJ software (Varian NMR Systems) and an Inova Agilent NMR spectrometer (Agilent Technologies Inc., Santa Clara, CA, USA), operating at a resonance frequency of ^1^H 600 MHz and a constant temperature of 298 K (25°C). A total of 256 free induction decays (FID) with 32-k data points over a spectral width of 8,000 Hz were used, with an acquisition time of 4 s and 1.5-s intervals between scans (relaxation delay). The spectral phase and baseline corrections, as well as the identification and quantification of the metabolites present in the samples, were performed using the Suite 7.6 Chenomx NMR software (Chenomx Inc., Edmonton, AB, Canada), with TMSP-d4 (concentration known) as a reference for quantifying the concentrations of other metabolites. All NMR spectra were processed with 0.5 Hz line broadening (lb) to smooth out the noise in the spectral signals. To inhibit any bias, the samples were randomly profiled by a blinded evaluator. Metabolites (methanol and ethanol) involved in the reagents used in the samples' collection and preparation were not considered for analysis. In addition, in relation to serum metabolites included in the study (Castro A., et al., 2019), the median within-test CV and ICC were 8.5% (range: 3–23%) and 0.98 (range: 0.79–1.00), respectively.

### Statistical Analysis

For all variables, the data distributions were checked for major deviation from normality. Logarithmic transformations (log_2_) were used to improve normality of distributions when appropriate (skewness values > 3.0). All transformed data were presented in their original scale for easier interpretation.

Pearson's correlation test was used to analyze the association of the changes (Δ, post-training values - pretraining values) in the metabolites' concentration levels and participant's physical characteristics with MPO gains. Afterwards, the ET and HIIT groups were fragmented, separately, into new subgroups, based on the first tercile (low responders, LRE) and third tercile (high responders, HRE) of the distribution of MPO gains in response to training (Castro A., et al., 2019). Comparisons at the pretraining and of the pre- to post-training changes between the LRE and HRE groups were performed using Student's *t-*test for independent samples. These analyses were performed using the PASW statistics software version 18.0 (SPSS, Chicago, IL), and the significance level adopted was 1% for these hypothesis-generating analyses, assuming that a Bonferroni correction would be too conservative, leading to a high rate of false-negative results. In addition, this approach was supplemented by estimating the effect size and 95% confidence interval for each comparison between LRE and HRE. Thus, when the confidence intervals did not cross zero, the differences were considered significant (Nakagawa and Cuthill, 2007). This approach helped to minimize the occurrence of type II error in the study.

For the identification of metabolic pathways altered by training associated with MPO gains, based on all correlational analyses performed, the metabolites that showed a nominal correlation of *r* ≥ |0.2| were retained for pathway over-representation and pathway topology analyses (Castro A., et al., 2019; Castro et al., 2021). Pathway analyses were based on the “Homo sapiens” library using Hypergeometric Test for Over Representation Analysis and Relative-Betweenness Centrality for Test Pathway Topology Analysis, as previously described (Xia and Wishart, 2010). The significance level was adjusted considering a false discovery rate of 0.1 (Benjamini and Ochberg, 1995) for the purpose of corrections due to multiple tests (van den Oord and Sullivan, 2003).

Finally, to determine the main altered metabolites associated with the MPO gains, only those supported by three levels of evidence were selected: (1) correlation with MPO gains (*r* ≥ |0.2|); (2) significant difference between HRE and LRE; and (3) contributing to significant metabolic pathways associated with MPO gains. Afterwards, multiple linear regression models with stepwise selection were used to determine the variance in MPO gains explained by the changes observed in each serum and/or skeletal muscle metabolites retained by the three levels of evidence and to point out the key metabolites. To validate the models, the assumption of multicollinearity of measures between the independent variables was assessed by the variance inflation factor (VIF 1); the normality of residue distribution was determined by inspecting the frequency histograms; and the global influence of each case in the model was analyzed by inspecting the standardized residues and Cook's distance (Field, 2009).

## Results

### Participants

The information regarding the correlations between the pretraining participant's characteristics and MPO gains were described in a previous publication (Castro A., et al., 2019). Briefly, there were no significant correlations between pretraining age, body mass, body fat percentage, BMI, and MPO with MPO gains for ET or HIIT programs (P > 0.01 for all).

### Association Between Changes in Metabolites Concentration Levels and MPO Gains

There were no significant correlations between MPO gains and pretraining to post-training changes in: body mass (ET: *r* = 0.26, *P* = 0.165, *n* = 30; HIIT: *r* = 0.22, *P* = 0.258, *n* = 30); body fat (ET: *r* = −0.17, *P* = 0.380, *n* = 30; HIIT: *r* = −0.04, *P* = 0.849, *n* = 29); and fat-free mass (ET: *r* = 0.29, *P* = 0.119, *n* = 30; HIIT: *r* = 0.36, *P* = 0.054, *n* = 29).

Of the 43 metabolites quantified in blood serum, 24 and 19 showed correlation coefficients (*r*) ≥ |0.2| for the association between MPO gains and the pretraining to post-training changes in metabolites' concentration levels of ET and HIIT programs, respectively (Table 1). For ET, the most correlated metabolites were glutamine, methionine, ornithine, creatine phosphate, and o-acetylcarnitine (*P* < 0.01 for all), asparagine, glycine, threonine, succinate, 3-hydroxybutyrate, xanthine, carnitine, and propylene glycol (*P* < 0.05 for all), while for HIIT, the most correlated were creatine (*P* < 0.01), guanidinoacetate and creatine phosphate (*P* < 0.05 for both). These correlations were moderate (ET: 0.39 ≤ *r* ≤ 0.64; HIIT: 0.45 ≤ *r* ≤ 0.54) and negative for all serum metabolites.

**Table 1:** Pearson's correlation coefficients (r) for the association between MPO gains and the pretraining to post–training changes in serum metabolites' concentration levels metabolic levels in the TIMES study.

**Serum metabolites^#^**	**ET** **(***n =*** 30)**	**HIIT** **(***n =*** 29)**
	**Δ MPO**	**Δ MPO**
***Amino acids***		
Alanine	−0.04	−0.06
Asparagine	−0.39^*^	−0.10
Glutamine	−0.51^**^	**−0.26**
Glycine	−0.41^*^	−0.14
Histidine	**−0.33**	0.02
Isoleucine	−0.16	**−0.31**
Lysine	−0.01	0.13
Methionine	−0.48^**^	0.16
Phenylalanine	**−0.20**	**0.23**
Proline	−0.03	−0.19
Threonine	−0.41^*^	−0.04
Tyrosine	−0.05	0.11
Valine	**−0.23**	**−0.31**
***Carboxylic Acids***		
Betaine	**−0.35**	**−0.20**
Creatinine	**−0.32**	0.05
Guanidinoacetate	**−0.34**	**−0.45** ^*^
N,N–Dimethylglycine	−0.18	−0.03
Ornithine	−0.50^**^	**−0.22**
Succinate	−0.39^*^	**−0.31**
Creatine	−0.09	**−0.54** ^**^
Creatine phosphate	−0.48^**^	**−0.48** ^*^
Formate	0.11	**0.29**
***Fatty acids***		
2–hydroxy–isocaproate	−0.18	**−0.24**
2–hydroxy–isovalerate	−0.05	−0.14
Methylsuccinate	−0.08	−0.07
O–Acetylcarnitine	−0.64^**^	**−0.20**
***Hydroxy Acids***		
3–hydroxybutyrate	−0.41^*^	**−0.31**
Lactate	0.04	0.05
Glycolate	**−0.32**	−0.02
***Imidazopyrimidines***		
Hypoxanthine	−0.16	−0.05
Xanthine	−0.41^*^	**−0.24**
***Organic Carbonic Acids***		
N–methylhydantoin	−0.07	−0.17^LT^
Urea	−0.08	**−0.23**
***Organic Oxygen Compounds***		
Glycerol	**−0.31**	−0.15
Carnitine	−0.42^*^	**−0.29**
Choline	**−0.30**	−0.01
Citrate	**−0.29**	0.03
Dimethyl sulfone	−0.05	−0.02
Trimethylamine	**−0.25**	**0.24**
Propylene glycol	−0.45^*^	**−0.21**
***Unclustered***		
Dimethylamine	−0.12	0.05
Inosine	−0.13	−0.14
Pyruvate	−0.12	−0.15

*ET, Endurance training; HIIT, High–intensity interval training; MPO, Maximal power output; Δ, post–training values – pretraining values*.

* *P < 0.05*.

** *P < 0.01. ^LT^Data log transformed before analysis. Values in bold are correlation coefficients (r) ≥ |0.2|*.

# *The metabolites' chemical taxonomy was based on the classes and subclasses of the Human Metabolome Database*.

Of the 70 metabolites quantified in the skeletal muscle, 42 and 18 showed correlation coefficients (*r*) ≥ |0.2| for the association between MPO gains and the pretraining to post-training changes in metabolites' concentration levels of ET and HIIT programs, respectively (Table 2). For ET, the most correlated metabolites were isobutyrate, histamine, pyruvate, AMP, glucose, histidine, 3-methylxanthine, ornithine, niacinamide (*P* < 0.01 for all), tyrosine, threonine, 2-phosphoglycerate, anserine, glycolate, glycine, N-nitrosodimethylamine, alanine, trimethylamine N-oxide, lactate and carnitine (*P* < 0.05 for all), while for HIIT, the most correlated were glycolate, nicotinate (*P* < 0.01 for both), histamine and 2-phosphoglycerate (*P* < 0.05 for both). These correlations were moderate (ET: 0.37 ≤ |*r*| ≤ 0.61; HIIT: 0.42 ≤ |*r*| ≤ 0.68) and negative for all these skeletal muscle metabolites, except for glycolate in both training programs and nicotinate in HIIT, which were positive.

**Table 2:** Pearson's correlation coefficients (r) for the association between MPO gains and the pretraining to post–training changes in skeletal muscle metabolites' concentration levels in the TIMES study.

**Skeletal muscle metabolites^#^**	**ET** **(***n =*** 29)**	**HIIT** **(***n =*** 28)**
	**Δ MPO**	**Δ MPO**
**Alcohols and Polyols**		
Ethylene glycol	−0.01	0.09
Myo–inositol	−0.15	−0.06
***Amino acids***		
Alanine	**−0.41** ^*^	0.03
Anserine	**−0.43** ^*^	0.09
Beta–Alanine	**−0.24** ^LT^	0.17
Glutamate	**−0.31** ^LT^	0.06
Glutamine	−0.19^LT^	0.09
Glycine	**−0.42** ^*^ ^LT^	0.05
Histidine	**−0.50** ^**^ ^LT^	0.16
Isoleucine	**−0.29**	−0.07
Leucine	**−0.21**	−0.17
Phenylalanine	**−0.35**	**0.21**
Proline	**−0.32** ^LT^	−0.16
Threonine	**−0.45** ^*^ ^LT^	0.11
Tyrosine	**−0.47** ^*^ ^LT^	−0.12
Valine	**0.34**	0.19
***Carboxylic Acids***		
Acetate	**−0.34**	−0.18
Betaine	0.05	0.19
Citrate	0.09	**0.36**
Creatine	**−0.26** ^LT^	−0.18
Creatine phosphate	0.08^LT^	**0.22**
Creatinine	**−0.28**	−0.11
Formate	−0.18 ^LT^	−0.09
Fumarate	**−0.33**	−0.14
Glutathione	−0.17	**0.25**
Isobutyrate	**−0.61** ^**^ ^LT^	**−0.36**
Isocitrate	**−0.21**	**0.23**
Maleate	0.08	0.11
Malonate	**−0.26** ^LT^	0.03
N,N–Dimethylglycine	−0.18	0.12
N–Acetylaspartate	−0.10	**0.21**
N–Acetylglutamine	−0.04	0.08
Nicotinate	**0.32**	**0.53** ^**^
Ornithine	**−0.48** ^**^	−0.16
Succinate	−0.17 ^LT^	0.09
π-Methyl–histidine	−0.13	0.02
τ-Methyl–histidine	0.06	**−0.35** ^LT^
***Fatty acids***		
2–Hydroxy–isocaproate	**−0.35** ^LT^	0.12
3–Hydroxy–isovalerate	−0.12^LT^	**0.36**
O–Acetylcarnitine	**−0.22** ^LT^	**−0.26**
***Hydroxy Acids***		
Glycolate	**0.42** ^*^	**0.68** ^**^
Lactate	**−0.39** ^*^	−0.08
***Imidazopyrimidines***		
3–Methylxanthine	**−0.48** ^**^	0.11
Oxipurinol	−0.08^LT^	0.11
Theophylline	**−0.33**	0.03
***Nucleosides and Nucleotides***		
ADP	−0.05	**0.22**
AMP	**−0.52** ^**^	−0.19
ATP	**−0.27** ^LT^	0.05
NAD+	−0.04	**0.27**
NADP+	**−0.22** ^LT^	0.11
***Organic Oxygen Compounds***	
2–Phosphoglycerate	**−0.44** ^*^	**−0.42** ^*^
Glucose	**−0.50** ^**^	−0.14
Glycerol	−0.01	−0.11
***Organic Nitrogen Compounds***		
Carnitine	**−0.37** ^*^	−0.06
Choline	0.19	0.16
Dimethylamine	**−0.32** ^LT^	0.13
Histamine	**−0.56** ^**^ ^LT^	**−0.43** ^*^
Methylamine	**−0.34** ^LT^	**−0.34**
N–Nitrosodimethylamine	**−0.42** ^*^	−0.15
Trimethylamine	**−0.31** ^LT^	0.07
Trimethylamine N–oxide	**−0.40** ^*^ ^LT^	0.18
Tartrate	0.19	0.12
***Unclustered***		
2–Hydroxyphenylacetate	**−0.31**	−0.10
Acetamide	−0.07	**0.38**
Carnosine	−0.14	−0.01
Dimethyl sulfone	−0.02	0.05
Niacinamide	**−0.47** ^**^	−0.16
Pyrimidine	**−0.20**	−0.04
Pyruvate	**−0.55** ^**^	0.00
Taurine	−0.03^LT^	0.02

*ET, Endurance training; HIIT, High–intensity interval training; MPO, Maximal power output; Δ, post–training values – pretraining values*.

* *P < 0.05*.

** *P < 0.01. ^LT^Data log transformed before analysis. Values in bold are correlation coefficients (r) ≥|0.2|*.

# *The metabolites' chemical taxonomy was based on the classes and subclasses of the Human Metabolome Database*.

### Differences Between Low and High Responders (LRE and HRE)

As demonstrated in previous study (Castro A., et al., 2019), there were no baseline differences between LRE and HRE for age, height, body mass, body fat, BMI, HR_MAX_ and MPO in the ET and HIIT programs (*P* > 0.01).

After ET, the pretraining to post-training changes for HRE showed a reduction in the serum levels of 3-hydroxybutyrate, asparagine, betaine, carnitine, citrate, creatine phosphate, creatinine, glutamine, glycine, glycolate, guanidinoacetate, histidine, methionine, O-acetylcarnitine, ornithine, propylene glycol, succinate, threonine, trimethylamine and xanthine, as well as lower increase in choline and glycerol levels compared to pretraining to post-training changes of LRE. The effect size (Cohen's *d*) for these comparisons was classified as wide, ranging from 0.99 to 1.98 (Table 3). In the skeletal muscle, the pretraining to post-training changes for HRE showed a higher reduction in the levels of histamine and isobutyrate, increase of glycolate and valine, and a reduction of 3-methylxanthine and pyruvate (effect size: 1.14 to 1.29) compared to LRE (Table 4).

**Table 3:** Comparison of the pretraining to post–training changes in serum metabolites' concentration levels between low responders (LRE) and high responders (HRE) to the ET and HIIT programs in the TIMES study.

**Metabolites (mM)^#^**	**ET**									**Metabolites (mM)^#^**	**HIIT**								
	**LRE** **(***n*** **=** 10)**			**HRE** **(***n*** **=** 10)**			**ES**	**95%**	**CI**		**LRE** **(***n*** **=** 9)**			**HRE** **(***n*** **=** 10)**			**ES**	**95%**	**CI**
3-Hydroxybutyrate	0.1124	±	0.1501	−0.0393	±	0.0889^*^	**1.23**	**0.22**	**2.24**	2-Hydroxy-isocaproate	0.0210	±	0.0341	−0.0002	±	0.0171	0.80	−0.19	1.79
Asparagine	0.0212	±	0.0210	−0.0030	±	0.0163^**^	**1.29**	**0.27**	**2.31**	3-Hydroxybutyrate	0.1011	±	0.2216	−0.0228	±	0.1942	0.60	−0.38	1.57
Betaine	0.0239	±	0.0293	−0.0076	±	0.0117^**^	**1.41**	**0.37**	**2.45**	Betaine	0.0197	±	0.0414	0.0039	±	0.0133	0.53	−0.44	1.50
Carnitine	0.0256	±	0.0280	−0.0051	±	0.0127^**^	**1.41**	**0.37**	**2.45**	Carnitine	0.0139	±	0.0211	−0.0037	±	0.0092^*^	**1.10**	**0.08**	**2.13**
Choline	0.0045	±	0.0027	0.0004	±	0.0033^**^	**1.35**	**0.33**	**2.38**	Creatine	0.0057	±	0.0102	−0.0118	±	0.0112^**^	**1.63**	**0.53**	**2.73**
Citrate	0.0504	±	0.0741	−0.0127	±	0.0256^*^	**1.14**	**0.14**	**2.14**	Creatine phosphate	0.0041	±	0.0059	−0.0022	±	0.0032^**^	**1.34**	**0.29**	**2.40**
Creatine phosphate	0.0023	±	0.0058	−0.0020	±	0.0020^*^	**1.00**	**0.01**	**1.98**	Formate	−0.0117	±	0.0112	0.0018	±	0.0155^*^	−0.98	−1.99	0.03
Creatinine	0.0563	±	0.0682	−0.0080	±	0.0262	**1.24**	**0.23**	**2.26**	Glutamine	0.1092	±	0.1504	0.0020	±	0.0804	0.90	−0.10	1.91
Glutamine	0.1128	±	0.1875	−0.1211	±	0.1757^**^	**1.29**	**0.27**	**2.31**	Guanidinoacetate	0.0138	±	0.0295	−0.0189	±	0.0209^*^	**1.29**	**0.24**	**2.34**
Glycerol	0.1507	±	0.1081	0.0012	±	0.0484^**^	**1.78**	**0.69**	**2.88**	Isoleucine	0.0242	±	0.0212	0.0047	±	0.0235	0.87	−0.13	1.87
Glycine	0.1584	±	0.1875	−0.0356	±	0.0988^**^	**1.29**	**0.28**	**2.31**	O–Acetylcarnitine	0.0043	±	0.0065	0.0011	±	0.0059	0.53	−0.44	1.50
Glycolate	0.0145	±	0.0175	−0.0021	±	0.0062^*^	**1.26**	**0.25**	**2.28**	Ornithine	0.0140	±	0.0241	0.0000	±	0.0198	0.64	−0.34	1.62
Guanidinoacetate	0.0304	±	0.0376	−0.0078	±	0.0205^*^	**1.26**	**0.25**	**2.28**	Phenylalanine	−0.0002	±	0.0143	0.0068	±	0.0146	−0.48	−1.45	0.48
Histidine	0.0344	±	0.0382	−0.0074	±	0.0268^*^	**1.27**	**0.25**	**2.28**	Propylene glycol	0.0041	±	0.0053	−0.0012	±	0.0043^*^	**1.09**	**0.07**	**2.12**
Methionine	0.0091	±	0.0099	−0.0039	±	0.0087^**^	**1.40**	**0.36**	**2.43**	Succinate	0.0057	±	0.0081	−0.0010	±	0.0027^*^	**1.13**	**0.11**	**2.16**
O–Acetylcarnitine	0.0029	±	0.0022	−0.0013	±	0.0020^**^	**1.98**	**0.85**	**3.11**	Trimethylamine	0.0001	±	0.0017	0.0006	±	0.0007	−0.45	−1.42	0.51
Ornithine	0.0267	±	0.0199	−0.0073	±	0.0213^**^	**1.65**	**0.58**	**2.73**	Urea	0.1478	±	0.2013	−0.0014	±	0.3778	0.49	−0.48	1.45
Phenylalanine	0.0136	±	0.0157	−0.0025	±	0.0179^*^	0.96	−0.02	1.93	Valine	0.1111	±	0.1066	0.0148	±	0.0557^*^	**1.15**	**0.12**	**2.18**
Propylene glycol	0.0051	±	0.0071	−0.0015	±	0.0042^*^	**1.14**	**0.14**	**2.14**	Xanthine	0.0030	±	0.0105	−0.0034	±	0.0097	0.63	−0.34	1.61
Succinate	0.0045	±	0.0077	−0.0037	±	0.0053^*^	**1.23**	**0.22**	**2.24**										
Threonine	0.0566	±	0.0664	−0.0214	±	0.0519^**^	**1.31**	**0.29**	**2.33**										
Trimethylamine	0.0015	±	0.0024	−0.0003	±	0.0011^*^	**0.99**	**0.01**	**1.98**										
Valine	0.1089	±	0.1515	0.0039	±	0.1110	0.79	−0.17	1.75										
Xanthine	0.0171	±	0.0128	−0.0033	±	0.0110^**^	**1.71**	**0.63**	**2.79**										

*The data are presented as mean ± standard deviation*.

*ET, Endurance training; HIIT, High–intensity interval training; LRE and HRE were stratified from the 1st and 3rd terciles, respectively, of the gains in maximal power output (MPO) in response to the ET and HIIT programs. ES, effect size (Cohen's d)*;

# *Metabolites with correlation coefficient (r) ≥ |0.2|for the association between MPO gains and pretraining to post–training changes in serum metabolites' concentration levels*.

** *P < **0.01** for independent t–test*.

* *P < **0.05** for independent t–test. Values in bold are ES and 95% CI that did not cross zero*.

**Table 4:** Comparison of the pretraining to post–training changes in skeletal muscle metabolites' concentration levels between low responders (LRE) and high responders (HRE) to the ET and HIIT programs in the TIMES study.

**Metabolites(mM.g^**−1**^)**	**ET**		**ES**			**Metabolites(mM.g^**−1**^)**	**HIIT**		**ES**		
	**LRE (*n =* 10)**	**HRE (*n =* 10)**		**95%**	**CI**		**LRE (*n =* 9)**	**HRE (*n =* 8)**		**95%**	**CI**
2–Hydroxy–isocaproate^LT^	−0.9767 ±0.0490	−1.1171 ± 0.2971	0.66	−0.29	1.61	2–Phosphoglycerate	−0.1949 ± 1.3958	−1.4458 ± 1.6225	0.83	−0.23	1.89
2–Hydroxyphenylacetate	−0.0044 ± 0.0217	−0.0291 ± 0.0634	0.52	−0.42	1.46	3–Hydroxy–isovalerate	0.0018 ± 0.0641	0.0530 ± 0.1377	−0.49	−1.52	0.54
2–Phosphoglycerate	0.2978 ± 1.4179	−2.1872 ± 3.4615	0.94	−0.04	1.92	ADP	−0.0067 ± 0.0154	0.0003 ± 0.0100	−0.53	−1.57	0.50
3–Methylxanthine	0.0045 ± 0.0525	−0.0701 ± 0.0756^*^	**1.15**	**0.15**	**2.15**	Acetamide	−0.0257 ± 0.0277	0.0049 ± 0.0240^*^	**−1.17**	**−2.27**	**−0.07**
Acetate	−0.0936 ± 0.1599	−0.3411 ± 0.4866	0.68	−0.27	1.64	Citrate	−0.0579 ± 0.1036	0.0472 ± 0.2108	−0.65	−1.69	0.40
Alanine	−0.1011 ± 0.6762	−1.3267 ± 2.3226	0.72	−0.24	1.67	Creatine phosphate	−4.9562 ± 4.9418	0.3420 ± 7.8008	−0.82	−1.88	0.24
AMP	0.0167 ± 0.0914	−0.0665 ± 0.0875	0.93	−0.05	1.91	Glutathione	−0.0468 ± 0.0691	0.0246 ± 0.1175	−0.75	−1.81	0.30
Anserine	0.0266 ± 0.0771	−0.0894 ± 0.2401	0.65	−0.30	1.60	Glycolate	−0.3983 ± 0.5146	0.5652 ± 0.7211^**^	**−1.56**	**−2.71**	**−0.40**
ATP^LT^	−1.0135 ± 0.0398	−1.0593 ± 0.1739	0.36	−0.57	1.30	Histamine	0.0045 ± 0.1524	−0.0952 ± 0.1977	0.57	−0.47	1.61
Beta–Alanine^LT^	−1.0054 ± 0.1144	−1.1512 ± 0.5371	0.38	−0.56	1.31	Isobutyrate	0.0591 ± 0.0677	−0.0264 ± 0.0557^*^	**1.37**	**0.24**	**2.50**
Carnitine	−0.3709 ± 0.9981	−1.2760 ± 1.4129	0.74	−0.22	1.70	Isocitrate	−0.0574 ± 0.2519	0.0590 ± 0.1322	−0.57	−1.60	0.47
Creatine^LT^	−0.0487 ± 3.0419	−1.4351 ± 4.0936	0.38	−0.55	1.32	Methylamine	0.0248 ± 0.0724	−0.0192 ± 0.0636	0.64	−0.40	1.69
Creatinine	−0.0081 ± 0.0789	−0.1002 ± 0.1285	0.86	−0.10	1.83	N–Acetylaspartate	−0.0224 ± 0.0273	−0.0070 ± 0.0236	−0.60	−1.64	0.44
Dimethylamine^LT^	−0.9836 ± 0.0227	−1.0350 ± 0.1187	0.60	−0.35	1.55	NAD+	0.0019 ± 0.0596	0.0666 ± 0.1037	−0.78	−1.83	0.28
Fumarate	0.0157 ± 0.0310	−0.0046 ± 0.0503	0.49	−0.45	1.43	Nicotinate	−0.0251 ± 0.0156	0.0081 ± 0.0157^*^	**−2.13**	**−3.40**	**−0.86**
Glucose	0.0424 ± 0.3745	−0.4060 ± 0.7525	0.75	−0.20	1.71	O–Acetylcarnitine	0.1582 ± 0.4713	−0.0505 ± 0.4058	0.47	−0.56	1.50
Glutamate^LT^	−0.4999 ± 0.7289	−1.0001 ± 1.3018	0.47	−0.47	1.41	Phenylalanine	−0.0310 ± 0.0225	−0.0151 ± 0.0561	−0.38	−1.41	0.64
Glycine^LT^	−1.1648 ± 0.4299	−1.7293 ± 0.9048	0.80	−0.17	1.76	τ–Methylhistidine^LT^	−1.1472 ± 0.6479	−1.7065 ± 1.0033	0.67	−0.37	1.72
Glycolate	−0.7410 ± 1.1241	0.5077 ± 0.8685^*^	**−1.24**	**−2.26**	**−0.23**						
Histamine^LT^	−0.7948 ± 0.2054	−1.2963 ± 0.5869	**1.14**	**0.14**	**2.14**						
Histidine^LT^	−0.9803 ± 0.1740	−1.3268 ± 0.6772	0.70	−0.25	1.66						
Isobutyrate^LT^	−0.8888 ± 0.0540	−1.2206 ± 0.3610	**1.29**	**0.27**	**2.30**						
Isocitrate	0.0247 ± 0.2148	−0.0472 ± 0.3212	0.26	−0.67	1.19						
Isoleucine	−0.0011 ± 0.0804	−0.1332 ± 0.2648	0.67	−0.28	1.63						
Lactate	0.2268 ± 2.4944	−1.8445 ± 3.5699	0.67	−0.28	1.63						
Leucine	−0.0388 ± 0.1024	−0.1232 ± 0.3741	0.31	−0.62	1.24						
Malonate^LT^	−1.2491 ± 0.4161	−1.5383 ± 0.7521	0.48	−0.46	1.42						
Methylamine^LT^	−0.9867 ± 0.0690	−1.0720 ± 0.2065	0.55	−0.39	1.50						
NADP+^LT^	−1.0010 ± 0.0193	−1.0132 ± 0.0625	0.26	−0.67	1.19						
Niacinamide	0.0049 ± 0.0600	−0.1276 ± 0.2090	0.86	−0.11	1.83						
Nicotinate	−0.0240 ± 0.0269	0.0090 ± 0.0472	−0.86	−1.83	0.11						
N–Nitrosodimethylamine	−0.0007 ± 0.0346	−0.0263 ± 0.0662	0.48	−0.46	1.42						
O–Acetylcarnitine^LT^	−1.0525 ± 0.6012	−1.2634 ± 1.1183	0.23	−0.70	1.16						
Ornithine	0.0163 ± 0.0762	−0.1099 ± 0.2270	0.74	−0.21	1.70						
Phenylalanine	−0.0098 ± 0.0241	−0.0586 ± 0.1284	0.53	−0.41	1.47						
Proline^LT^	−0.9875 ± 0.2060	−1.5528 ± 1.2327	0.64	−0.31	1.59						
Pyrimidine	0.0066 ± 0.0182	−0.0014 ± 0.0423	0.24	−0.69	1.17						
Pyruvate	0.0499 ± 0.1795	−0.1651 ± 0.1892^*^	**1.17**	**0.16**	**2.17**						
Theophylline	−0.0069 ± 0.1430	−0.1247 ± 0.2248	0.63	−0.32	1.57						
Threonine^LT^	−0.9830 ± 0.2012	−1.3190 ± 0.5785	0.78	−0.19	1.74						
Trimethylamine^LT^	−1.0079 ± 0.0484	−1.0532 ± 0.1455	0.42	−0.52	1.36						
Trimethylamine N–oxide^LT^	−1.0195 ± 0.1255	−1.2582 ± 0.4373	0.74	−0.22	1.70						
Tyrosine^LT^	−0.9753 ± 0.0480	−1.0891 ± 0.1656	0.93	−0.04	1.91						
Valine	−0.2042 ± 0.0899	0.0741 ± 0.1140^**^	**−2.71**	**−4.00**	**−1.43**						

*The data are presented as mean ± standard deviation*.

*ET, Endurance training; HIIT, High–intensity interval training; LRE and HRE were stratified from the 1st and 3rd terciles, respectively, of the gains in maximal power output (MPO) in response to the ET and HIIT programs. ES, effect size (Cohen's d; # Metabolites with correlation coefficient (r) ≥ |0.2|for the association between MPO gains and pretraining to post–training changes in skeletal muscle metabolites' concentration levels*.

** *P < **0.01** for independent t–test*.

* *P < **0.05** for independent t–test. “Values in bold are ES and 95% CI that did not cross zero*.

After HIIT, the pretraining to post-training changes for HRE showed a reduction in the serum levels of carnitine, creatine, creatine phosphate, guanidinoacetate, propylene glycol and succinate, and a lower increase in valine levels compared to LRE. The effect size for these comparisons was classified as wide, ranging from 1.09 to 1.63 (Table 3). In the skeletal muscle, HRE showed an increase in the levels of acetamide, glycolate and nicotinate (effect size: −1.17 to −2.27) and a reduction of isobutyrate levels (effect size: 1.37) compared to LRE (Table 4).

### Metabolic Pathways

**Figure 1:**
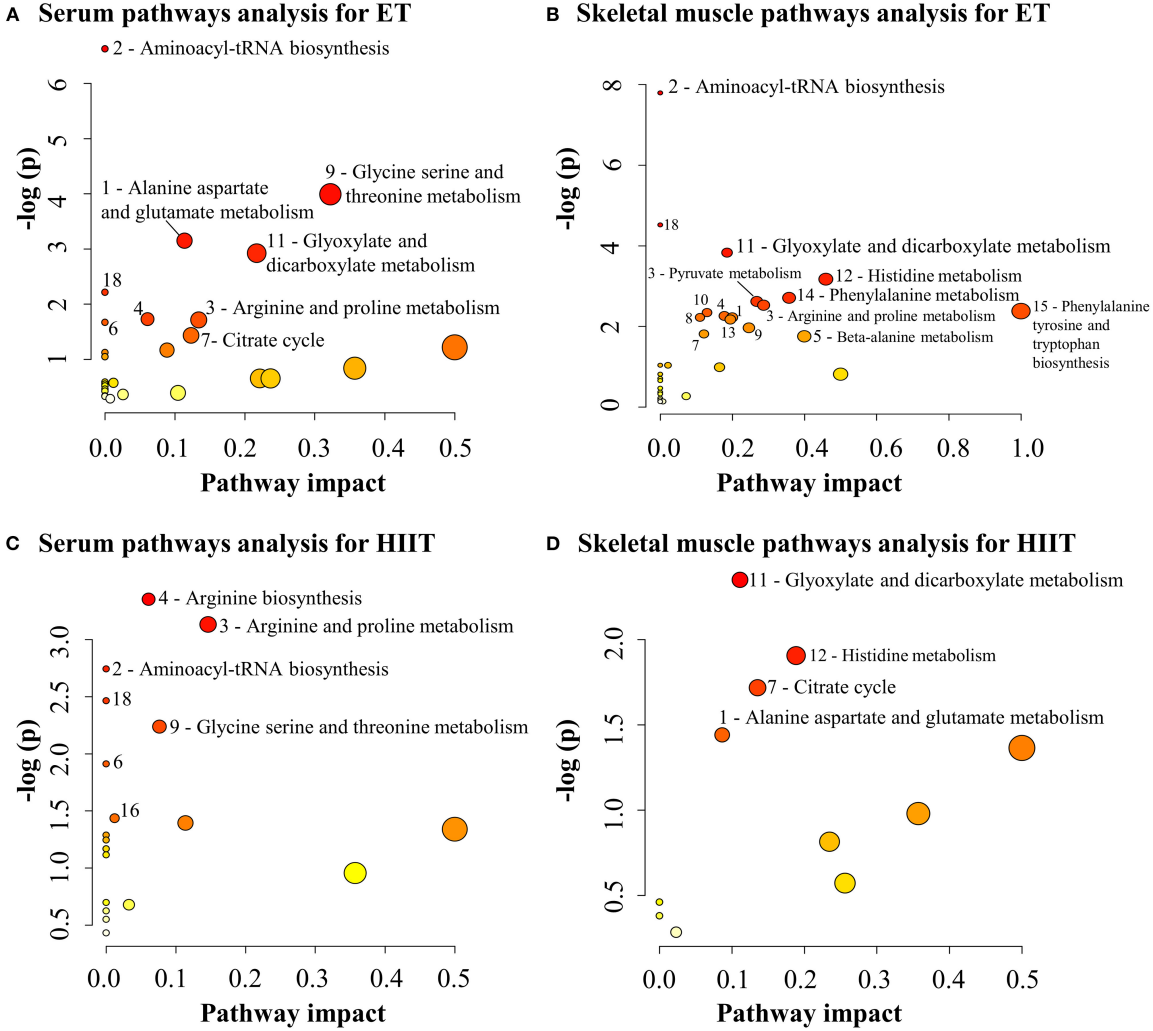
Summary of altered metabolic pathways and their metabolites associated with maximal power output gains after endurance training (ET) and high intensity interval training (HIIT). The numbers and labels in the figures represent the most enriched and impacted pathways. All numbered pathways have significance for a false discovery rate of 0.1 (vertical axis). The pathway's impact on the horizontal axis represents the relative contribution of all identified metabolites in relation to those that compose it. (1) Alanine, aspartate and glutamate metabolism (**A**: asparagine, glutamine, citrate and succinate; **B**: alanine, pyruvate, glutamine and fumarate; **C**: glutamine and succinate; **D**: N-acetylaspartate and citrate); (2) Aminocyl-tRNA biosynthesis (**A**: asparagine, histidine, phenylalanine, glutamine, glycine, methionine, valine and threonine; **B**: histidine, phenylalanine, glycine, alanine, isoleucine, leucine, threonine, tyrosine, proline, valine and glutamate; **C**: phenylalanine, glutamine, valine and isoleucine); (3) Arginine and proline metabolism (**A**: ornithine, guanidinoacetate, creatine phosphate; **B**: ornithine, glutamate, proline, creatine, and pyruvate; **C**: ornithine, guanidinoacetate, creatine, and creatine phosphate); (4) Arginine biosynthesis (**A**: ornithine and glutamine; **B**: glutamate, ornithine, and fumarate; **C**: ornithine, glutamine, and urea); (5) Beta-alanine metabolism (**B**: beta-alanine, anserine and histidine); (6) Butanoate metabolism (**B**, **C**: 3-hydroxybutyrate and succinate); (7) Citric acid cycle (**A**: succinate and citrate; **B**: isocitrate, pyruvate and fumarate; **D**: isocitrate and citrate); (8) Glutathione metabolism (**B**: glycine, glutamate, NADP+ and ornithine); (9) Glycine, serine and threonine metabolism (**A**: choline, betaine, guanidinoacetate, glycine and threonine; **B**: glycine, threonine, creatine and pyruvate; **C**: betaine, guanidinoacetate and creatine); (10) Glycolysis or gluconeogenesis (**B**: pyruvate, lactate, glucose and acetate); (11) Glyoxalate and dicarboxylate metabolism (**A**: glycolate, citrate, glycine, and glutamine; **B**: glycolate, glycine, glutamate, acetate, isocitrate, and pyruvate; **C**: isocitrate, glycolate and citrate); (12) Histidine metabolism (**B**: glutamine, histidine, anserine and histamine; **D**: anserine and histamine); (13) Nicotinate and nicotinamide metabolism (**B**: nicotinamide, NADP+, and nicotinate); (14) Phenylalanine metabolism (**B**: phenylalanine, 2-hydroxyphenylacetate and tyrosine); (15) Phenylalanine tyrosine and tryptophan biosynthesis (**B**: phenylalanine and tyrosine); (16) Purine metabolism (**C**: xanthine and glutamine); (17) Pyruvate metabolism (**B**: pyruvate, lactate, fumarate and acetate); (18) Valine, leucine and isoleucine biosynthesis (**A**: threonine and valine; **B**: threonine, leucine, isoleucine, and valine; **C**: valine and isoleucine).

For pathway analysis, pretraining to post-training changed metabolites that were correlated at *r* ≥ |0.2|with MPO gains were used, separately for serum (ET: 24 metabolites; HIIT: 19 metabolites) and skeletal muscle (ET: 42 metabolites; HIIT: 18 metabolites) in each exercise program. A total of 18 distinct and significantly changed pathways were identified and related to MPO gains, at a false discovery rate of 0.1. From these pathways, only 6 in serum (aminoacyl-tRNA biosynthesis, arginine and proline metabolism, arginine biosynthesis, butanoate metabolism, glycine serine and threonine metabolism, and valine leucine and isoleucine biosynthesis) and 4 in skeletal muscle (alanine aspartate and glutamate metabolism, citrate cycle, glyoxylate and dicarboxylate metabolism, histidine metabolism) were similar between ET and HIIT programs. The complete list of significant pathways and their related metabolites for each training program are summarized in detail in Supplementary Table 1 and Figure 1.

### Summary of Key Altered Metabolites Associated With MPO Gains

The altered metabolites associated with gains in MPO were identified based on three levels of evidence, previously described: (1) correlation with MPO gains (*r* ≥ |0.2|); (2) significant differences between LRE and HRE; and (3) contribution in significant altered pathways related to MPO gains.

The metabolites supported by the three levels of evidence in the ET program were: asparagine, glutamine, succinate, glycine, histidine, methionine, threonine, creatine phosphate, guanidinoacetate, ornithine, citrate, 3-hydroxybutyrate, betaine, choline and glycolate in blood serum; and pyruvate, glycolate, valine and histamine in the skeletal muscle. On the other hand, in the HIIT program, they were: succinate, valine, creatine, creatine phosphate, and guanidinoacetate in blood serum; and glycolate in the skeletal muscle (Table 5).

**Table 5:** Summary of altered metabolites and their metabolic pathways associated with MPO gains in response to ET and HIIT, supported by the three levels of evidence in the TIMES study.

**Metabolic pathways^#^**	**ET**		**HIIT**		**Reference Metabolism**
	**Serum**	**Muscle**	**Serum**	**Muscle**	
Alanine, aspartate and glutamate metabolism	Asparagine, glutamine, citrate, and succinate	Pyruvate	Succinate		Amino acid metabolism
Aminoacyl–tRNA biosynthesis	Asparagine, histidine, glutamine, glycine, methionine, threonine, and valine	Valine	Valine		Translational process
Arginine and proline metabolism	Guanidinoacetate, ornithine, and creatine phosphate	Pyruvate	Guanidinoacetate, creatine phosphate and creatine		Amino acid metabolism
Arginine biosynthesis	Ornithine and glutamine				Amino acid metabolism
Butanoate metabolism	3–hydroxybutyrate and succinate		Succinate		Carbohydrate metabolism
Citrate cycle	Succinate and citrate	Pyruvate			Carbohydrate metabolism
Glycine, serine and threonine metabolism	Choline, betaine, guanidinoacetate, glycine, and threonine	Pyruvate	Guanidinoacetate and creatine		Amino acid metabolism
Glycolysis/Gluconeogenesis		Pyruvate			Carbohydrate metabolism
Glyoxylate and dicarboxylate metabolism	Glycolate, citrate, glycine, and glutamine	Glycolate and pyruvate		Glycolate	Carbohydrate metabolism
Histidine metabolism		Histamine			Amino acid metabolism
Pyruvate metabolism		Pyruvate			Carbohydrate metabolism
Valine, leucine and isoleucine biosynthesis	Threonine	Valine	Valine		Amino acid metabolism

*MPO, Maximal power output; ET, Endurance training; HIIT, High–intensity interval training*.

# *Based on the Kyoto Encyclopedia of Genes and Genomes (KEGG) Pathway Database*.

From these metabolites, multiple linear regression models were conducted in order to determine the true interindividual response variance of the MPO gains (changes free from the effects caused by the intervention and the changes that would have occurred in the absence of intervention) explained by each metabolite supported by the three levels of evidence (Table 6). For ET, the variance in MPO gains was explained: 77.4% by the intervention effects; 6.9, 2.3, 3.2, and 2.2% by changes in skeletal muscle pyruvate and valine, serum glutamine and creatine phosphate, respectively. For HIIT, the variance in MPO gains was explained: 80.9% by the intervention effects; 7.2, 2.2, and 1.2% by changes in skeletal muscle glycolate, serum creatine and creatine phosphate, respectively.

**Table 6:** Results of the multivariate linear regression model with stepwise selection for the MPO gains in response to training in the TIMES Study.

**Models**	**β**	**B (95% CL)**		**F–value**	**Probability > F**	**r^**2**^ Change**	**r^**2**^ Model**	**VIF**
*Model 1*								
Control	Reference			———	———	———	———	———
ET	0.89	59.6	(52.4; 66.8)	126.5	<0.001	0.774	0.774	1.17
Skeletal muscle pyruvate	−0.19	−30.9	(−48.8; −13.1)	96.2	<0.001	0.069	0.842	1.22
Serum glutamine	−0.16	−26.1	(−43.4; −8.7)	81.5	<0.001	0.032	0.875	1.08
Serum creatine phosphate	−0.17	−1,132.1	(−1,851.3; −412.9)	74.1	<0.001	0.022	0.897	1.10
Skeletal muscle valine	0.16	16.3	(5.7; 27.0)	76.3	<0.001	0.023	0.920	1.09
*Model 2*								
Control	Reference			———	———	———	———	———
HIIT	0.83	60.3	(52.1; 68.5)	144.3	<0.001	0.809	0.809	1.12
Skeletal muscle glycolate	0.21	10.0	(4.4; 15.6)	122.1	<0.001	0.072	0.881	1.25
Serum creatine	−0.12	−217.4	(−414.4; −20.3)	98.8	<0.001	0.022	0.903	1.10
Serum creatine phosphate	−0.12	−870.7	(−1720.3; −21.2)	83.0	<0.001	0.012	0.915	1.19

*MPO, Maximum power output; ET, Endurance training; HIIT, High–intensity interval training; β, Standardized coefficient; B, Unstandardized coefficient; VIF, Variance inflation fator*.

## Discussion

This study investigated whether changes in the metabolic profile and metabolic pathways of blood serum and the skeletal muscle are associated with the trainability of CRF, based on MPO, in response to ET and HIIT programs. The results were based on the commonality of three levels of evidence and the main findings were: (i) differences in the metabolic changes associated with the MPO gains between training programs, as well as between LRE and HRE; (ii) associations between changes in the metabolic profile and MPO gains, which were: negative for serum asparagine, glutamine, succinate, glycine, histidine, methionine, threonine, creatine phosphate, guanidinoacetate, ornithine, citrate, 3-hydroxybutyrate, betaine, choline, glycolate, and skeletal muscle pyruvate and histamine in the ET program; negative for serum succinate, valine, creatine, creatine phosphate, and guanidinoacetate in the HIIT program; and positive for skeletal muscle valine in ET and glycolate in both ET and HIIT programs; (iii) identification of key altered metabolites that were able to explain the interindividual response variance of the MPO gains, adjusted by random errors and intervention effects: 14.7%, based on changes of skeletal muscle pyruvate and valine, serum glutamine and creatine phosphate in the ET program; 10.5%, based on changes of skeletal muscle glycolate, serum creatine and creatine phosphate in the HIIT program (Table 6); (iv) the most impacted pathways (impact > 0) by these key altered metabolites were: arginine and proline metabolism, glycine, serine and threonine metabolism, and glyoxylate and dicarboxylate metabolism for both ET and HIIT programs; alanine, aspartate and glutamate metabolism, arginine biosynthesis, glycolysis/gluconeogenesis, and pyruvate metabolism for ET (Table 5).

In order to summarize the results, only the key metabolites, supported by the three levels of evidence, will be discussed. In the case of ET, these metabolites were serum glutamine and creatine phosphate, skeletal muscle pyruvate and valine. Glutamine is produced from the reaction of ammonia with glutamate, being responsible for the transfer of nitrogen between organs or for the synthesis of nucleotides, detoxification of ammonia and maintenance of the acid-base balance in the kidneys, in addition to serving as fuel for immune cells and signaling the regulation of protein synthesis and degradation (Pérez-Sala et al., 1987; Hood and Terjung, 1990; Newsholme et al., 2003). Previous studies have shown higher circulating levels of glutamine in athletes compared to sedentary people and after endurance training (Kargotich et al., 2007), which were however lower in athletes with *overtraining* (Rowbottom et al., 1995), pointing to glutamine reduction as an indicator of training overload (Rowbottom et al., 1995, 1996). Conversely, in the present study, there was a negative association of changes in serum glutamine with MPO gains. This can be attributed to the greater degradation of amino acids during prolonged fasting in LRE individuals, increasing the supply of amine groups and the production of ammonia, precursors of glutamine production. This hypothesis can be supported in part by the inverse relationship observed also for essential amino acids such as valine, threonine and histidine, and for the enrichment of the metabolic pathway of alanine, glutamate and aspartate.

Creatine phosphate, on the other hand, is a molecule that stores energy within the muscle and promotes the immediate replenishment of ATP during intense exercise. Although circulating creatine phosphate levels have rarely been reported (Harris et al., 2004; Kalim et al., 2015), the present study identified low concentrations (4–5 μM) of this metabolite in the circulation, which, when changed by training, were negatively associated with the trainability of CRF in both ET and HIIT programs. Although the reasons for this result are not clear, it is possible to speculate on the occurrence of cell damage promoted by the sum of consecutive sessions performed in the last week of training (Baird et al., 2012; Sureda et al., 2015) as a mechanism related to the extravasation of the circulating creatine phosphate. This hypothesis is based on previous studies demonstrating evidence of cell damage due to the increase in creatine kinase observed after up to eight wk of aerobic training (De Araujo et al., 2013), as well as 24–48 h after successive sessions of acute aerobic exercise (Baird et al., 2012), which corroborate with the adopted timing of blood collection in the present study. Unfortunately, the creatine kinase or creatine phosphate levels (quantified by other methods) were not measured in this study, otherwise it would be possible to confirm or refute this hypothesis.

Similarly, in the skeletal muscle, changes in pyruvate were negatively associated with MPO gains. This result corroborates findings from other studies that demonstrated a reduction in pyruvate levels concomitantly with an increase in CRF after moderate to vigorous aerobic training (Henderson et al., 2004; LeBlanc et al., 2004; Huffman et al., 2014). The attenuated production of pyruvate at rest after training has been attributed to the improvement in the cellular energy supply (availability of free ADP and AMP, and inorganic phosphate) and decrease in the glycogenolysis rate, mediated by the decrease in the activity of the pyruvate dehydrogenase complex, which is responsible for decarboxylating pyruvate and supplying the citric acid cycle with Acetyl-CoA (LeBlanc et al., 2004; Han et al., 2020). Additionally, it is likely that the reduction in the amount of Acetyl-CoA supplied by pyruvate and via glycogenolysis is being offset by the amount derived from the oxidation of fatty acids (Nelson and Cox, 2013). In accordance with these results, there was also a positive association with changes in valine levels, an essential branched-chain amino acid (BCAA) which is required for protein synthesis in the skeletal muscle (Harper et al., 1984) and MPO gains. The results found for both pyruvate and valine in the skeletal muscle suggest that HRE individuals may benefit from a more efficient mechanism of fatty acid oxidation and muscle protein synthesis with aerobic training (Overmyer et al., 2015; Li et al., 2018).

For HIIT, the key metabolites were serum creatine and creatine phosphate, and skeletal muscle glycolate. Creatine is synthesized in the liver and kidneys from guanidinoacetate, derived from glycine and arginine, then it is then released into the circulation and transported to the skeletal muscle, where it will be stored as creatine phosphate serve as a source of rapid ATP production during high-intensity exercise (Walker, 1979). In this sense, there is evidence that the increased availability of circulating creatine is associated with an improvement in CRF indicators after HIIT programs (Graef et al., 2009; Kendall et al., 2009). However, in the present study, the increase in creatine levels was demonstrated concomitantly with the increase in its guanidinoacetate precursor, suggesting an imbalance in creatine metabolism (Walker, 1979) and possibly partially explaining the negative association with changes in CRF shown by both. Another point that should be highlighted is that high-intensity exercise can promote changes in renal functions (Bellinghieri et al., 2008). Thus, given that the kidneys are the main producers of guanidinoacetate (Edison et al., 2007), monitoring renal function markers may prove to be useful for understanding the trainability of CRF in future studies.

Additionally, changes in the skeletal muscle glycolate, involved in glyoxalate and dicarboxylate metabolism, were positively associated with MPO gains. Glycolate is a glyoxalate precursor that produces oxaloacetate, an intermediate in the citric acid cycle (Miao et al., 2018). Although the relationship between muscle glycolate and the adaptations induced by exercise or aerobic training in humans is not widely known (Castro A., et al., 2019; Danaher et al., 2020), previous studies with animal models corroborate the results obtained here, showing greater activation of glyoxalate and dicarboxylate metabolism in trained rats compared to sedentary ones (Starnes et al., 2017), as well as in rats with high CRF compared to those with low CRF (Falegan et al., 2017), in addition to a positive association with increased fatigue resistance in rats submitted to exhaustive aerobic exercise (Miao et al., 2018). It is tempting to speculate that increased levels of glycolate may be associated with an improved citrate cycle activity, via oxaloacetate production derived from glyoxalate and dicarboxylate metabolism, perhaps contributing to MPO gains regulation. In this sense, recently studies have shown associations between baseline glyoxalate and dicarboxylate metabolism activity with intrinsic (Castro et al., 2021) and acquired MPO levels (Castro A., et al., 2019).

In summary, our results demonstrated that the inter-individual variability of CRF in responses to ET and HIIT programs seems to be primarily associated with the individual's potential to regulate fasting energy supply through amino acid and carbohydrate metabolism. As we observed, there was a decrease in metabolites indicatives of pyruvate metabolism and glycolysis metabolism pretraining to post-training, as well as of amino acid metabolism (arginine and proline metabolism, glycine, serine and threonine metabolism, alanine, aspartate and glutamate metabolism, and arginine biosynthesis), while an increase in metabolites precursor of intermediates of the citric acid cycle via glyoxylate and dicarboxylate metabolism was found.

Some important limitations to present study should be highlighted. Both training programs tested consisted of eight wk of training, which is generally not sufficient to achieve the maximum response to a given dose of exercise (Ross et al., 2015b). Thus, it is possible that the specific time needed to achieve the physiological adaptations in each training program (Astorino et al., 2013; Ross et al., 2015b; MacInnis and Gibala, 2017; O'Connor and Malone, 2019) contributed to the differences observed in the association between mechanisms related to the trainability of CRF between ET and HIIT. Diet was not controlled during the entire experimental period. Since the participants were not hospitalized, this type of control would be almost impossible; however, all participants were constantly asked to avoid major changes in nutritional habits, such as, changes that would lead to substantial fluctuations in body weight. Despite this, it is important to highlight that the variability of the interindividual response of CRF or other biochemical health markers is expected to happen regardless of diet (Bouchard et al., 2012; Ross et al., 2015a,b). In addition, the results of the present study are limited to two types of aerobic training, so generalizations to other programs or different intensities should be avoided, since variations in individual responses can be protocol or dose-dependent (Huffman et al., 2014; Bonafiglia et al., 2016; Joyner and Lundby, 2018; Williams et al., 2019). It is important to consider that the moment of biopsy and blood collection, 48 h after the last training session, may not represent the optimal moment to investigate the chronic changes induced by training in all identified metabolites. We also recognize that our tertile-based classification of exercise responders will by default result in 33% low and 33% high responders. In this sense, these classification terminologies must be taken with caution when comparing studies, as they reflect the distribution of MPO gains values and context in the population of the TIMES study. Most of previous studies have investigated the integrative mechanisms of variability of individual CRF responses measured by maximal oxygen uptake, but not necessarily referencing MPO, which makes it difficult to compare with our findings. However, MPO as a surrogate of CRF is known to present low technical error and high test-retest reproducibility (Skinner et al., 1999; Montero and Lundby, 2017). The TIMES study cohort consists of healthy and sedentary young Caucasian men which potentially limiting the generalizability of our results. Replication studies are warranted. Lastly, some strengths of the present study are that the results were based essentially on the commonality among three levels of evidence minimizing the occurrence of metabolites occasionally associated to the MPO gains. Blood and muscle tissue were collected at fasting state after a prior 12 h diet control. The explained variance of the MPO gains was interpreted under the changes in key metabolites adjusted by the effects caused by the intervention and absence of intervention (random error) as previously recommended (Ross et al., 2019).

## Conclusion

This study has demonstrated distinct serum and skeletal muscle metabolites between ET and HIIT programs, who's pretraining to post-training changes associated with the inter-individual variability of CRF responses. Additionally, the panel of pretraining to post-training changed metabolites also indicated some similar pathways between ET and HIIT associated with variability of CRF responses, suggesting the involvement of amino acid and carbohydrate metabolism. These results provide new insights to investigate the underlying changes in metabolism that are determinant for inter-individual variability of CRF in responses to ET and HIIT programs.

## Data Availability Statement

The original contributions presented in the study are included in the article/Supplementary Material, further inquiries can be directed to the corresponding author/s.

## Ethics Statement

The studies involving human participants were reviewed and approved by University of Campinas' Research Ethics Committee (Number: 2.717.688; CAAE: 52997216.8.0000.5404). The patients/participants provided their written informed consent to participate in this study.

## Author Contributions

AC and MC-M conceptualized and designed the study. AC, RD, and SO-N conducted training program, experimental data collection, and metabolomics analysis. AA performed the muscle biopsies. MC-M and CC provided technical assistance and/or conceptual advice. AC performed statistical analysis and wrote the first draft of the manuscript. All authors have read, edited and approved the submitted version.

## Funding

MC-M is supported by grants from the São Paulo Research Foundation (FAPESP, No. 2016/057417) and Support Fund for Teaching, Research and Extension (FAEPEX, No. 2021/16). AC also was supported by the National Council for Scientific and Technological Development (CNPq, No. 149201/2015-0), Coordination for the Improvement of Higher Education Personnel (PDSE-CAPES, No. 88881.135219/2016-01), and São Paulo Research Foundation (FAPESP, No. 2020/13939-7).

## Conflict of Interest

The authors declare that the research was conducted in the absence of any commercial or financial relationships that could be construed as a potential conflict of interest.

## Publisher's Note

All claims expressed in this article are solely those of the authors and do not necessarily represent those of their affiliated organizations, or those of the publisher, the editors and the reviewers. Any product that may be evaluated in this article, or claim that may be made by its manufacturer, is not guaranteed or endorsed by the publisher.
