# Supplementary material for: Association Between Changes in Serum and Skeletal Muscle Metabolomics Profile With Maximum Power Output Gains in Response to Different Aerobic Training Programs: The Times Study
Source: Front Physiol. 2021 Oct 20;12:756618. doi: 10.3389/fphys.2021.756618 (PMC8563999; doi:10.3389/fphys.2021.756618)
Supplement: Supplementary file 1 [file Table_1.DOCX]

| **Supplementary Table 1**. Summary of serum and skeletal muscle pathways for both training programs in the TIMES Study. | | | | | | | | | | | |
| --- | --- | --- | --- | --- | --- | --- | --- | --- | --- | --- | --- |
| **Sample** | **Group** | **Pathway** | **Total** | **Expected** | **Hits** | **Raw p** | **-LOG10(p)** | **Impact** | **P-value Ranking** | **FDR** | **Decision** |
| Muscle | ET | Aminoacyl-tRNA biosynthesis | 48 | 1.30 | 11 | 0.0000 | 7.79 | 0.00 | 1 | 0.0011 | SIG |
| Serum | ET | Aminoacyl-tRNA biosynthesis | 48 | 0.74 | 8 | 0.0000 | 6.63 | 0.00 | 2 | 0.0022 | SIG |
| Muscle | ET | Valine leucine and isoleucine biosynthesis | 8 | 0.22 | 4 | 0.0000 | 4.52 | 0.00 | 3 | 0.0033 | SIG |
| Serum | ET | Glycine serine and threonine metabolism | 33 | 0.51 | 5 | 0.0001 | 3.99 | 0.32 | 4 | 0.0044 | SIG |
| Muscle | ET | Glyoxylate and dicarboxylate metabolism | 32 | 0.87 | 6 | 0.0001 | 3.83 | 0.19 | 5 | 0.0056 | SIG |
| Serum | HIIT | Arginine biosynthesis | 14 | 0.16 | 3 | 0.0004 | 3.35 | 0.06 | 6 | 0.0067 | SIG |
| Muscle | ET | Histidine metabolism | 16 | 0.43 | 4 | 0.0007 | 3.17 | 0.46 | 7 | 0.0078 | SIG |
| Serum | ET | Alanine aspartate and glutamate metabolism | 28 | 0.43 | 4 | 0.0007 | 3.15 | 0.11 | 8 | 0.0089 | SIG |
| Serum | HIIT | Arginine and proline metabolism | 38 | 0.44 | 4 | 0.0007 | 3.13 | 0.15 | 9 | 0.0100 | SIG |
| Serum | ET | Glyoxylate and dicarboxylate metabolism | 32 | 0.50 | 4 | 0.0012 | 2.92 | 0.22 | 10 | 0.0111 | SIG |
| Serum | HIIT | Aminoacyl-tRNA biosynthesis | 48 | 0.56 | 4 | 0.0018 | 2.74 | 0.00 | 11 | 0.0122 | SIG |
| Muscle | ET | Phenylalanine metabolism | 10 | 0.27 | 3 | 0.0019 | 2.71 | 0.36 | 12 | 0.0133 | SIG |
| Muscle | ET | Pyruvate metabolism | 22 | 0.60 | 4 | 0.0024 | 2.62 | 0.27 | 13 | 0.0144 | SIG |
| Muscle | ET | Arginine and proline metabolism | 38 | 1.03 | 5 | 0.0030 | 2.53 | 0.29 | 14 | 0.0156 | SIG |
| Serum | HIIT | Valine leucine and isoleucine biosynthesis | 8 | 0.09 | 2 | 0.0034 | 2.47 | 0.00 | 15 | 0.0167 | SIG |
| Muscle | ET | Phenylalanine, tyrosine and tryptophan biosynthesis | 4 | 0.11 | 2 | 0.0042 | 2.38 | 1.00 | 16 | 0.0178 | SIG |
| Muscle | HIIT | Glyoxylate and dicarboxylate metabolism | 32 | 0.35 | 3 | 0.0045 | 2.35 | 0.11 | 17 | 0.0189 | SIG |
| Muscle | ET | Glycolysis/gluconeogenesis | 26 | 0.70 | 4 | 0.0045 | 2.34 | 0.13 | 18 | 0.0200 | SIG |
| Muscle | ET | Arginine biosynthesis | 14 | 0.38 | 3 | 0.0055 | 2.26 | 0.18 | 19 | 0.0211 | SIG |
| Serum | HIIT | Glycine serine and threonine metabolism | 33 | 0.38 | 3 | 0.0058 | 2.24 | 0.08 | 20 | 0.0222 | SIG |
| Muscle | ET | Glutathione metabolism | 28 | 0.76 | 4 | 0.0060 | 2.23 | 0.11 | 21 | 0.0233 | SIG |
| Muscle | ET | Alanine aspartate and glutamate metabolism | 28 | 0.76 | 4 | 0.0060 | 2.23 | 0.20 | 22 | 0.0244 | SIG |
| Serum | ET | Valine leucine and isoleucine biosynthesis | 8 | 0.12 | 2 | 0.0061 | 2.22 | 0.00 | 23 | 0.0256 | SIG |
| Muscle | ET | Nicotinate and nicotinamide metabolism | 15 | 0.41 | 3 | 0.0067 | 2.17 | 0.19 | 24 | 0.0267 | SIG |
| Muscle | ET | Glycine serine and threonine metabolism | 33 | 0.89 | 4 | 0.0108 | 1.97 | 0.25 | 25 | 0.0278 | SIG |
| Serum | HIIT | Butanoate metabolism | 15 | 0.17 | 2 | 0.0122 | 1.91 | 0.00 | 26 | 0.0289 | SIG |
| Muscle | HIIT | Histidine metabolism | 16 | 0.18 | 2 | 0.0124 | 1.91 | 0.19 | 27 | 0.0300 | SIG |
| Muscle | ET | Citrate cycle (tca cycle) | 20 | 0.54 | 3 | 0.0153 | 1.82 | 0.12 | 28 | 0.0311 | SIG |
| Muscle | ET | Beta-alanine metabolism | 21 | 0.57 | 3 | 0.0175 | 1.76 | 0.40 | 29 | 0.0322 | SIG |
| Serum | ET | Arginine biosynthesis | 14 | 0.22 | 2 | 0.0187 | 1.73 | 0.06 | 30 | 0.0333 | SIG |
| Muscle | HIIT | Citrate cycle (tca cycle) | 20 | 0.22 | 2 | 0.0192 | 1.72 | 0.14 | 31 | 0.0344 | SIG |
| Serum | ET | Arginine and proline metabolism | 38 | 0.59 | 3 | 0.0193 | 1.71 | 0.13 | 32 | 0.0356 | SIG |
| Serum | ET | Butanoate metabolism | 15 | 0.23 | 2 | 0.0213 | 1.67 | 0.00 | 33 | 0.0367 | SIG |
| Muscle | HIIT | Alanine aspartate and glutamate metabolism | 28 | 0.31 | 2 | 0.0362 | 1.44 | 0.09 | 34 | 0.0378 | SIG |
| Serum | HIIT | Purine metabolism | 65 | 0.75 | 3 | 0.0366 | 1.44 | 0.01 | 35 | 0.0389 | SIG |
| Serum | ET | Citrate cycle (tca cycle) | 20 | 0.31 | 2 | 0.0368 | 1.43 | 0.12 | 36 | 0.0400 | SIG |
| Serum | HIIT | Alanine aspartate and glutamate metabolism | 28 | 0.33 | 2 | 0.0403 | 1.39 | 0.11 | 37 | 0.0411 | SIG |
| Muscle | HIIT | Phenylalanine tyrosine and tryptophan biosynthesis | 4 | 0.04 | 1 | 0.0432 | 1.36 | 0.50 | 38 | 0.0422 | NS |
| Serum | HIIT | Phenylalanine tyrosine and tryptophan biosynthesis | 4 | 0.05 | 1 | 0.0457 | 1.34 | 0.50 | 39 | 0.0433 | NS |
| Serum | HIIT | Glyoxylate and dicarboxylate metabolism | 32 | 0.37 | 2 | 0.0514 | 1.29 | 0.00 | 40 | 0.0444 | NS |
| Serum | HIIT | Synthesis and degradation of ketone bodies | 5 | 0.06 | 1 | 0.0568 | 1.25 | 0.00 | 41 | 0.0456 | NS |
| Serum | ET | Phenylalanine tyrosine and tryptophan biosynthesis | 4 | 0.06 | 1 | 0.0606 | 1.22 | 0.50 | 42 | 0.0467 | NS |
| Serum | HIIT | D-glutamine and d-glutamate metabolism | 6 | 0.07 | 1 | 0.0678 | 1.17 | 0.00 | 43 | 0.0478 | NS |
| Serum | HIIT | Nitrogen metabolism | 6 | 0.07 | 1 | 0.0678 | 1.17 | 0.00 | 44 | 0.0489 | NS |
| Serum | ET | Glutathione metabolism | 28 | 0.43 | 2 | 0.0680 | 1.17 | 0.09 | 45 | 0.0500 | NS |
| Serum | ET | Synthesis and degradation of ketone bodies | 5 | 0.08 | 1 | 0.0752 | 1.12 | 0.00 | 46 | 0.0511 | NS |
| Serum | HIIT | Valine leucine and isoleucine degradation | 40 | 0.46 | 2 | 0.0766 | 1.12 | 0.00 | 47 | 0.0522 | NS |
| Serum | ET | D-glutamine and d-glutamate metabolism | 6 | 0.09 | 1 | 0.0895 | 1.05 | 0.00 | 48 | 0.0533 | NS |
| Serum | ET | Nitrogen metabolism | 6 | 0.09 | 1 | 0.0895 | 1.05 | 0.00 | 49 | 0.0544 | NS |
| Muscle | ET | Valine leucine and isoleucine degradation | 40 | 1.08 | 3 | 0.0914 | 1.04 | 0.00 | 50 | 0.0556 | NS |
| Muscle | ET | Pantothenate and coa biosynthesis | 19 | 0.51 | 2 | 0.0917 | 1.04 | 0.02 | 51 | 0.0567 | NS |
| Muscle | ET | Tyrosine metabolism | 42 | 1.14 | 3 | 0.1023 | 0.99 | 0.16 | 52 | 0.0578 | NS |
| Muscle | HIIT | Phenylalanine metabolism | 10 | 0.11 | 1 | 0.1047 | 0.98 | 0.36 | 53 | 0.0589 | NS |
| Serum | HIIT | Phenylalanine metabolism | 10 | 0.12 | 1 | 0.1106 | 0.96 | 0.36 | 54 | 0.0600 | NS |
| Serum | ET | Phenylalanine metabolism | 10 | 0.15 | 1 | 0.1449 | 0.84 | 0.36 | 55 | 0.0611 | NS |
| Muscle | ET | Nitrogen metabolism | 6 | 0.16 | 1 | 0.1522 | 0.82 | 0.00 | 56 | 0.0622 | NS |
| Muscle | ET | D-glutamine and d-glutamate metabolism | 6 | 0.16 | 1 | 0.1522 | 0.82 | 0.50 | 57 | 0.0633 | NS |
| Muscle | HIIT | Nicotinate and nicotinamide metabolism | 15 | 0.16 | 1 | 0.1531 | 0.82 | 0.23 | 58 | 0.0644 | NS |
| Muscle | ET | Porphyrin and chlorophyll metabolism | 30 | 0.81 | 2 | 0.1941 | 0.71 | 0.00 | 59 | 0.0656 | NS |
| Serum | HIIT | Pantothenate and coa biosynthesis | 19 | 0.22 | 1 | 0.2001 | 0.70 | 0.00 | 60 | 0.0667 | NS |
| Serum | HIIT | Citrate cycle (tca cycle) | 20 | 0.23 | 1 | 0.2095 | 0.68 | 0.03 | 61 | 0.0678 | NS |
| Muscle | ET | Ubiquinone and other terpenoid-quinone biosynthesis | 9 | 0.24 | 1 | 0.2196 | 0.66 | 0.00 | 62 | 0.0689 | NS |
| Serum | ET | Histidine metabolism | 16 | 0.25 | 1 | 0.2219 | 0.65 | 0.22 | 63 | 0.0700 | NS |
| Serum | ET | Glycerolipid metabolism | 16 | 0.25 | 1 | 0.2219 | 0.65 | 0.24 | 64 | 0.0711 | NS |
| Serum | HIIT | Propanoate metabolism | 23 | 0.27 | 1 | 0.2371 | 0.63 | 0.00 | 65 | 0.0722 | NS |
| Serum | ET | Pantothenate and coa biosynthesis | 19 | 0.29 | 1 | 0.2579 | 0.59 | 0.00 | 66 | 0.0733 | NS |
| Serum | ET | Purine metabolism | 65 | 1.01 | 2 | 0.2665 | 0.57 | 0.01 | 67 | 0.0744 | NS |
| Muscle | HIIT | Glutathione metabolism | 28 | 0.31 | 1 | 0.2677 | 0.57 | 0.26 | 68 | 0.0756 | NS |
| Serum | ET | Beta-alanine metabolism | 21 | 0.33 | 1 | 0.2810 | 0.55 | 0.00 | 69 | 0.0767 | NS |
| Serum | HIIT | Glutathione metabolism | 28 | 0.33 | 1 | 0.2811 | 0.55 | 0.00 | 70 | 0.0778 | NS |
| Serum | ET | Propanoate metabolism | 23 | 0.36 | 1 | 0.3034 | 0.52 | 0.00 | 71 | 0.0789 | NS |
| Muscle | ET | Butanoate metabolism | 15 | 0.41 | 1 | 0.3390 | 0.47 | 0.00 | 72 | 0.0800 | NS |
| Muscle | HIIT | Arginine and proline metabolism | 38 | 0.42 | 1 | 0.3457 | 0.46 | 0.00 | 73 | 0.0811 | NS |
| Serum | ET | Galactose metabolism | 27 | 0.42 | 1 | 0.3462 | 0.46 | 0.00 | 74 | 0.0822 | NS |
| Serum | HIIT | Pyrimidine metabolism | 39 | 0.45 | 1 | 0.3695 | 0.43 | 0.00 | 75 | 0.0833 | NS |
| Serum | ET | Porphyrin and chlorophyll metabolism | 30 | 0.46 | 1 | 0.3766 | 0.42 | 0.00 | 76 | 0.0844 | NS |
| Serum | ET | Cysteine and methionine metabolism | 33 | 0.51 | 1 | 0.4057 | 0.39 | 0.10 | 77 | 0.0856 | NS |
| Muscle | HIIT | Aminoacyl-trna biosynthesis | 48 | 0.53 | 1 | 0.4159 | 0.38 | 0.00 | 78 | 0.0867 | NS |
| Muscle | ET | Selenocompound metabolism | 20 | 0.54 | 1 | 0.4247 | 0.37 | 0.00 | 79 | 0.0878 | NS |
| Serum | ET | Glycerophospholipid metabolism | 36 | 0.56 | 1 | 0.4335 | 0.36 | 0.03 | 80 | 0.0889 | NS |
| Serum | ET | Pyrimidine metabolism | 39 | 0.60 | 1 | 0.4600 | 0.34 | 0.00 | 81 | 0.0900 | NS |
| Serum | ET | Valine leucine and isoleucine degradation | 40 | 0.62 | 1 | 0.4686 | 0.33 | 0.00 | 82 | 0.0911 | NS |
| Muscle | ET | Propanoate metabolism | 23 | 0.62 | 1 | 0.4708 | 0.33 | 0.00 | 83 | 0.0922 | NS |
| Serum | ET | Primary bile acid biosynthesis | 46 | 0.71 | 1 | 0.5174 | 0.29 | 0.01 | 84 | 0.0933 | NS |
| Muscle | HIIT | Purine metabolism | 65 | 0.71 | 1 | 0.5191 | 0.28 | 0.02 | 85 | 0.0944 | NS |
| Muscle | ET | Purine metabolism | 65 | 1.76 | 2 | 0.5336 | 0.27 | 0.07 | 86 | 0.0956 | NS |
| Muscle | ET | Cysteine and methionine metabolism | 33 | 0.89 | 1 | 0.6000 | 0.22 | 0.00 | 87 | 0.0967 | NS |
| Muscle | ET | Pyrimidine metabolism | 39 | 1.06 | 1 | 0.6621 | 0.18 | 0.00 | 88 | 0.0978 | NS |
| Muscle | ET | Primary bile acid biosynthesis | 46 | 1.25 | 1 | 0.7227 | 0.14 | 0.01 | 89 | 0.0989 | NS |
| Muscle | ET | Fatty acid biosynthesis | 47 | 1.27 | 1 | 0.7304 | 0.14 | 0.00 | 90 | 0.1000 | NS |
| FDR: false discovery rate of 0.1. All identified pathways were ranked based on their p-values from smallest to largest. Then, the FDR values were calculated for each pathway (q), assuming q = (0.1˟i)/*m*, where i denotes the i-*th* position of the pathway in the ranking of p-values (in the i-*th* row) and *m* the total number of ranked pathways. When the p-values of the pathways were less than the q-values, the pathway was declared to be significantly enriched; SIG: Significantly enriched pathway at the FDR level; NS: Non-significantly enriched pathway at the FRD level. | | | | | | | | | | | |
